# Magnetic Pendulum Arrays for Efficient ULF Transmission

**DOI:** 10.1038/s41598-019-49341-4

**Published:** 2019-09-13

**Authors:** Srinivas Prasad M N, Rustu Umut Tok, Foad Fereidoony, Yuanxun Ethan Wang, Rui Zhu, Adam Propst, Scott Bland

**Affiliations:** 1 Department of Electrical and Computer Engineering, University of California, Los Angeles, USA; 2 AxEnd Inc, Los Angeles, USA; 3 NextGen Aeronautics, Torrance, USA

**Keywords:** Electrical and electronic engineering, Mechanical engineering

## Abstract

The frequencies lying between 300 Hz to 3 kHz have been designated as Ultra Low Frequency (ULF) with corresponding wavelengths from 1000 Km to 100 Km. Although ULF has very low bandwidth it is very reliable, penetrating and difficult to jam which makes it a great choice for communication in underwater and underground environments. Small and portable ULF antennas within a diameter of 1 meter would operate under an electrical length on the order of 10^−4^ to 10^−6^ wavelengths in free space, making them very inefficient because of fundamental limits on radiation from electrically small antennas. To overcome this problem, Mechanical Antennas or ‘Mechtennas’ for Ultra Low Frequency Communications have been proposed recently. For efficient generation of ULF radiation, we propose a portable electromechanical system called a Magnetic Pendulum Array (MPA). A proof of concept demonstration of the system at 1.03 kHz is presented. The theory and experimental results demonstrate that such a system can achieve a significantly higher quality factor than conventional coils and thus order of magnitude higher transmission efficiency. The concept can be easily scaled to the ULF range of frequencies.

## Introduction

The use of conventional high frequency radio transmission and reception is impractical for underwater and underground communications as the environment can distort and, in many cases, completely block the signals from propagating between the transmitter and receiver. The large attenuation of the high frequency electromagnetic fields can be explained from the skin depth (*δ*) equation given by $\delta =\frac{1}{\sqrt{\pi f\mu \sigma }}$, where *f* is the frequency of the field, *μ* is the magnetic permeability of the material and *σ* is the conductivity of the material^1^. We can observe that the skin depth for a 100 MHz signal is about three orders of magnitude smaller as compared to the skin depth of a 1 kHz signal in the same medium. Thus, it is advantageous to use low frequency signals when the medium has strong absorption. Frequencies lying between 300 Hz to 3 kHz have been designated as Ultra Low Frequency (ULF), with corresponding wavelengths from 1000 Km to 100 Km. Radio signals at these frequencies can penetrate some distance into media such as water, soil, and rock. Although ULF has limited bandwidth, and thus low data rates, it is an attractive choice for underwater and underground environments where high frequency radio signals cannot reach as ULF is reliable, can penetrate these media, and difficult to interfere with. ULF is advantageous over the use of acoustic waves because it is immune from the reverberation associated with acoustic waves, especially in and around the obstacles such as bridges and vessel hulls. Therefore, ULF has been widely used for military applications involving long range communications with submarines and underground mines.

At present, constructing ULF antennas is very costly because the wavelengths at these frequencies range from a few kms to hundreds of kms. It is well known that the size of the antenna is proportional to the wavelength at which it operates, so communications at ULF has resulted in the construction of gigantic antennas which consume MWs of power. Any practical electrically small antenna would be very inefficient because the radiated power is now a tiny fraction of the stored energy on the antenna according to Chu’s limit^2^ while the dissipated power is usually a much greater fraction of the same energy given by the finite quality factor of the material that comprises the antenna. The former ratio can be increased by reducing the radiation quality factor, i.e., increasing the electrical dimension of the antenna. The U.S. Navy’s Very Low Frequency (VLF, 3–30 kHz) antenna in Cutler, Maine occupies 2000 acres on a peninsula and consists of 26 towers, each 850 to 1000 ft high. It consumes 18 MW power from a dedicated power plant^3^. On the other hand, the radiation efficiency can also be enhanced with the use of a higher quality factor transmitting antenna^4^. A space-borne antenna for ULF/VLF radiation has been described in^5^. It consists of a superconducting DC rotating coil surrounded by magnetoplasma operating as a ULF transmitter. It has been shown that such a space-borne antenna is not engineeringly feasible^5^. A novel phased orthogonal loop antenna is used to create a rotating magnetic field source, which creates low frequency waves called Alfven waves (80 to 355 kHz) as described in^6^. It requires a high power LRC driver circuit with peak-to-peak current of 1200 A and voltages across the capacitors of 2000 V.

A novel method of using the mechanical motion of charges to produce ULF radiation has recently gained momentum. Several mechanical antennas, or ‘mechtennas’, have been proposed in^7–12^, and these generate low frequency radiation. The difference between a conventional electrically small antenna and a mechtenna lies in the fact that conventional antennas create dynamic electromagnetic fields by relying on field-accelerated charges, whereas in mechtennas radiated time-varying fields are generated by physically moving, rotating or oscillating the electric charges or magnetic dipole moments. The radiation efficiency of a conventional electrically small antenna is limited by its ohmic loss, which is the dominating loss mechanism. It will be shown that such ohmic loss can be overcome by mechanical antennas. The key for high efficiency mechanical antennas lies in the development of a high-quality factor (high - Q) mechanical oscillatory system that has extremely small damping and effectively couples to the changing dipole moments. Most of the proposed mechtennas utilize either rotating electric or magnetic dipoles or piezo electric materials driven at acoustic resonance. for generating low frequency radiation. The rotating systems require a great amount of mechanical energy to be injected before the antenna rotates at the desired speed. The piezo electric systems have been demonstrated at Very Low Frequency (VLF) range. This work focuses on an innovative mechanically driven transmitter not consisting of rotating magnets or piezo electric materials but rather consists of an array of magnetic pendulums in oscillatory motion at ULF. There are several merits when a pendulum-based system over a rotating magnet based mechtenna is used. The first merit is that in a pendulum the stored energy and the mechanical motion in such a system is built up gradually at a constant frequency. The pendulums can form an array easily without the need for maintaining axially symmetry. Consequently, the total effective magnetic dipole moment remains the same while stored energy per unit volume reduces as the dimension of each pendulum reduces^13^. The third advantage is that the stored energy in a pendulum system alternates between two states, namely the kinetic energy due to inertia and the magnetic potential energy due to the magnetic field re-distribution. This allows one to control the motion and to enforce modulation to the radiation through the so-called Direct Antenna Modulation (DAM) approach^14^. Finally, the pendulums require no electric motor to drive and the only dominant loss mechanism in the proposed magnetic pendulum array is the friction of the pendulum supporting bearings. Hence, one can achieve a very high-Q factor in such a system that will greatly enhance the transmission efficiency for near-field ULF communications.

## Methods and Results

### Proposed system design with magnetic pendulum array

Mechanical antennas, as mentioned earlier, rely on physically moving electric charges or magnetic dipole moments through oscillatory mechanical movements to generate a dynamic electromagnetic field, which avoids the ohmic loss of a conventional conductor-based antenna. Mechanical antennas based on rotating magnets have been proposed in^7–11^. A spinning magnetic dipole field can be represented by superposition of two orthogonal magnetic dipole solutions in space with a 90-degree phase^10,15^. Assuming, the rotation is with an angular frequency *ω*, the following electromagnetic fields can be obtained for an oscillating magnetic dipole at a distance *r*:

1 $$\overrightarrow{E}=\frac{{\mu }_{o}{m}_{o}sin\theta }{4\pi r}[\frac{{\omega }^{2}}{c}\,\cos (\omega (t-\frac{r}{c}))+\frac{\omega }{r}\,\sin (\omega (t-\frac{r}{c}))]\hat{\varphi }$$

2 $$\overrightarrow{B}=\frac{{\mu }_{o}{m}_{o}cos\theta }{2\pi {r}^{2}}[\frac{1}{r}\,\cos (\omega (t-\frac{r}{c}))-\frac{\omega }{c}\,\sin (\omega (t-\frac{r}{c}))]\hat{r}+\frac{{\mu }_{o}{m}_{o}sin\theta }{4\pi r}[(\frac{1}{{r}^{2}}-\frac{{\omega }^{2}}{{c}^{2}})\cos (\omega (t-\frac{r}{c}))-\frac{\omega }{rc}\,\sin (\omega (t-\frac{r}{c}))]\hat{\theta }$$

In the far field we can obtain the following expressions for the radiated fields ${\overrightarrow{E}}_{rad}$ and ${\overrightarrow{B}}_{rad}$ for a rotating magnetic dipole assuming the observer is much further away than the wavelength ($r\gg c/\omega$)

3 $${\overrightarrow{B}}_{rad}=\frac{{\mu }_{o}{m}_{o}{\omega }^{2}}{4\pi r{c}^{2}}[\cos (\omega (t-\frac{r}{c}))(\hat{x}-\frac{x}{r}\hat{r})+\,\sin (\omega (t-\frac{r}{c}))(\hat{y}-\frac{y}{r}\hat{r})]$$

4 $${\overrightarrow{E}}_{rad}=-\,c(\hat{r}\times {\overrightarrow{B}}_{rad})$$

where ${\mu }_{o}$ is the permeability of free space, ${m}_{o}$ is the magnetic dipole moment and c is the speed of light in vacuum. Therefore, the spinning magnet will generate a circularly polarized electromagnetic wave in the far field at the direction normal to the rotation plane or linear polarization in the plane of rotation that can be used for far-field communications. In the near field, the magnetic field component dominates, and one may obtain the near field by rotating the magnetic dipole field expression according to the rotational axis^15^, yielding

5 $$\overrightarrow{B}=\frac{{\mu }_{o}{m}_{o}cos\theta }{2\pi {r}^{3}}[\cos (\omega (t-\frac{r}{c}))]\hat{r}+\frac{{\mu }_{o}{m}_{o}sin\theta }{4\pi {r}^{3}}[\cos (\omega (t-\frac{r}{c}))]\hat{\theta }$$

**Figure 1:**
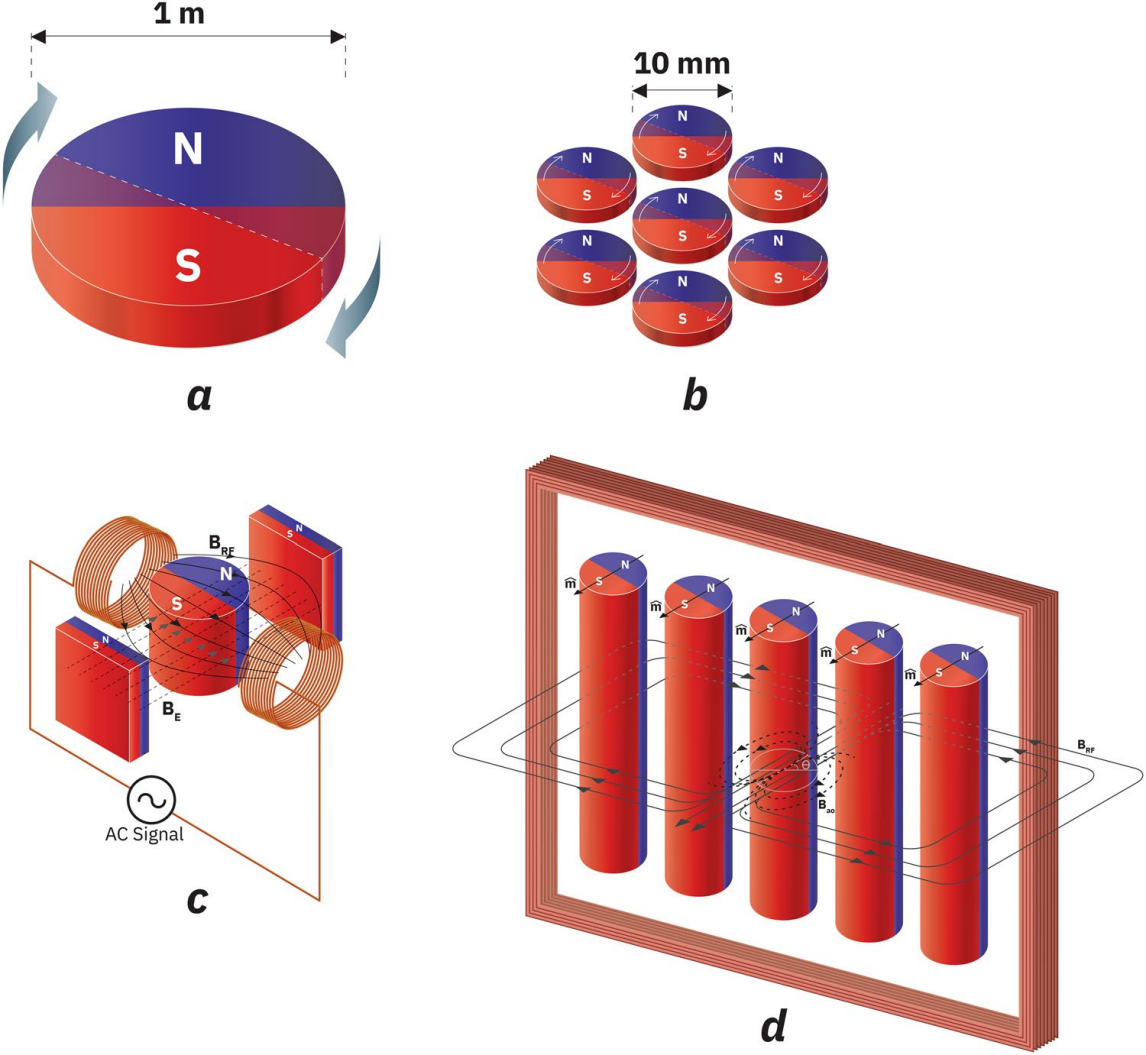
Evolution of Self Biased Magnetic Pendulum Array. (**a**) Spinning magnet driven by external motor. (**b**) An array of spinning magnets driven by external motors. (**c**) Externally biased magnetic pendulum driven by an external AC field. (**d**) Self-biased magnetic pendulum array driven by and external AC field.

It is evident that in the near field the longitudinal B field component can be best used for ULF communications. The unique characteristic of spinning magnet as shown in Fig. 1(a) is that the magnetic near field has the stored electromagnetic energy which does not require pairing with another form of energy and is constant irrespective of the angular frequency of rotation and orientation. We can ignore the mechanical energy required for spinning the magnet if the magnetization to mass ratio is high enough. But there are many difficulties associated with such a system. Practically speaking, transmitting ULF signals at 1 kHz would mean physically rotating the magnets at the speed of 60,000 rpm, which is not trivial from a mechanical design perspective. The system must be designed in a such way that the frictional energy losses are minimized, and the tensile stress limits are met. An even more fundamental issue associated with the spinning magnet system is that the dynamic magnetic energy created can get overwhelmed by the mechanical energy required to spin the magnet to designated speed. Introduction of modulation of angular velocity would require replenishment of the energy difference which might get dissipated between the two modulation states leading to lower efficiency of the system. The total mechanical energy *W*_*ME*_ associated with a circular disc of radius *r*, mass *m* and volume V, rotating at an angular velocity *ω* can be written as

6 $${W}_{ME}=\frac{1}{2}I{\omega }^{2}$$

where $I=\frac{m{r}^{2}}{2}$ is the moment of inertia of the magnets.

We can write the average energy density as

7 $$\frac{{W}_{ME}}{V}=\frac{1}{4}{\omega }^{2}{r}^{2}\rho $$

For a circular Neodymium based magnet with a radius of 0.5 m and mass density 7500 *kg*/*m*^3^ spinning at 1 kHz, the approximate mechanical energy density on an average is 1.85 × 10^7^ *KJ*/*m*^3^. In comparison, the typical energy density of an NdFeB magnet obtained from its *BH*_*max*_ value is around 470 *kJ*/*m*^3^ ^16^. The five orders of magnitude energy density difference in a spinning magnet implies that most of the energy in the system remains in the mechanical domain rather than the magnetic domain, thus is inefficient in creating electromagnetic coupling or radiation. One could make a case for a spinning magnet array consisting of an array of synchronously rotating smaller magnets which can not only emulate a larger spinning disc in its magnetic field behavior but also lower the mechanical energy density significantly, as shown in Fig. 1(b). The challenge is that the original axial symmetry of the system is broken which would create energy peaks and wells at different angles that prohibit an energy free rotation. The efficiency of a motor driving these magnets will also be problematic as it is at free running conditions. Any further reduction in size of the motor driven spinning magnet will also be impractical from the manufacturing perspective due to the prohibitive complexity. Modulating spinning magnets is difficult since all the parts are in a constant, high angular velocity motion.

To realize an ULF mechtenna with high radiation efficiency, a high-Q mechanical system that is easy to control is necessary. Pendulums are well-known to be high-Q and likewise a magnetic pendulum consisting of a diametrically magnetized cylinder and a pair of external magnets shown in Fig. 1(c) is one of such high-Q systems. The external magnets would align the cylinder to a direction of south to north and north to south and any disturbance to that equilibrium condition with external excitation will create an oscillatory swinging motion of the cylindrical magnet. The idea can be further extended to an array of such magnetic pendulums in smaller diameters which not only reduces the mechanical energy density of the system but also eliminates the need for external biasing magnets. As shown in Fig. 1(d), the system consists of cylindrical magnets which are diametrically magnetized and arranged in an array fashion. The array is said to be self-biased as a static external magnetic field is not required for their alignment. The alignment of each magnet in the array to the array axis occurs by itself due to the field of the adjacent magnets. An external coil is placed around the housing in the plane of the magnets to excite the magnets to create a time-varying magnetic field *B*_*RF*_ that is perpendicular to the pendulum array plane, which can physically rotate the magnets’ magnetic orientation towards the out of plane direction. In general, the magnitude of the radio frequency magnetic field is much lower than the magnitude of DC magnetic field, but in a high Q system the RF magnetic field can substantially deviate the magnets away from their lowest energy state eventually, as oscillatory motion is built up given enough excitation time^13^. The RF coil injects the RF energy that is not only needed to build up the oscillatory motion but also to replenish any losses in the form of friction loss or eddy current loss, including the RF energy that is radiated and dissipated after the magnetic pendulum array reaches its steady state^13^. The frictional and eddy current losses can be minimized to be an extremely small fraction of energy in the system with proper design. A dynamic magnetic field is created in the out of plane direction due to the oscillatory motion of the magnets^13^ which can be used for near field communication and energy transfer. We hypothesize that such a self-biased magnetic pendulum array can create 100% mechanical-to-magnetic coupling and a high Q vibration of the magnetic field for efficient ULF transmission. In the following section we calculate the resonant frequency of the magnetic pendulum array.

### Resonance frequency calculation

The angular acceleration of a mass is governed by

8 $$\frac{{d}^{2}\theta }{d{t}^{2}}I=-\,T$$

where I is the angular moment of inertia and T is a torque on the rotating mass. A pendulum with cylindrical shape has a moment of inertia

9 $${\rm{I}}=\frac{{m}_{a}{r}^{2}}{2}$$

where *m*_*a*_ is the mass and *r* is the radius of the cylinder. This differential equation results in a second order system with oscillatory solutions when the torque is a function of angular position. The magnetic pendulum receives a magnetic torque $\overrightarrow{T}$ given by

10 $$\overrightarrow{T}=\overrightarrow{m}\times \overrightarrow{B}$$

where $\overrightarrow{m}$ is the magnetic dipole moment and $\overrightarrow{B}$ is the magnetic field, which can be an external field added through a biasing magnet or an internal field created by the neighboring pendulums.

For a single pendulum in a uniform external magnetic field *B*_*e*_, the pendulum equation can be shown as:

11 $$\frac{{d}^{2}\theta }{d{t}^{2}}=-\,\frac{2{M}_{s}{B}_{e}}{\rho {r}^{2}}\theta $$

where *M*_*s*_ is the saturation magnetization and $\rho$ is the mass density. The solution to the swing angle *θ* is given by

12 $${\rm{\theta }}({\rm{t}})={{\rm{\theta }}}_{{\rm{\max }}}\,\sin (\frac{1}{r}\sqrt{\frac{2{M}_{s}{B}_{e}}{\rho }}t)$$

**Figure 2:**
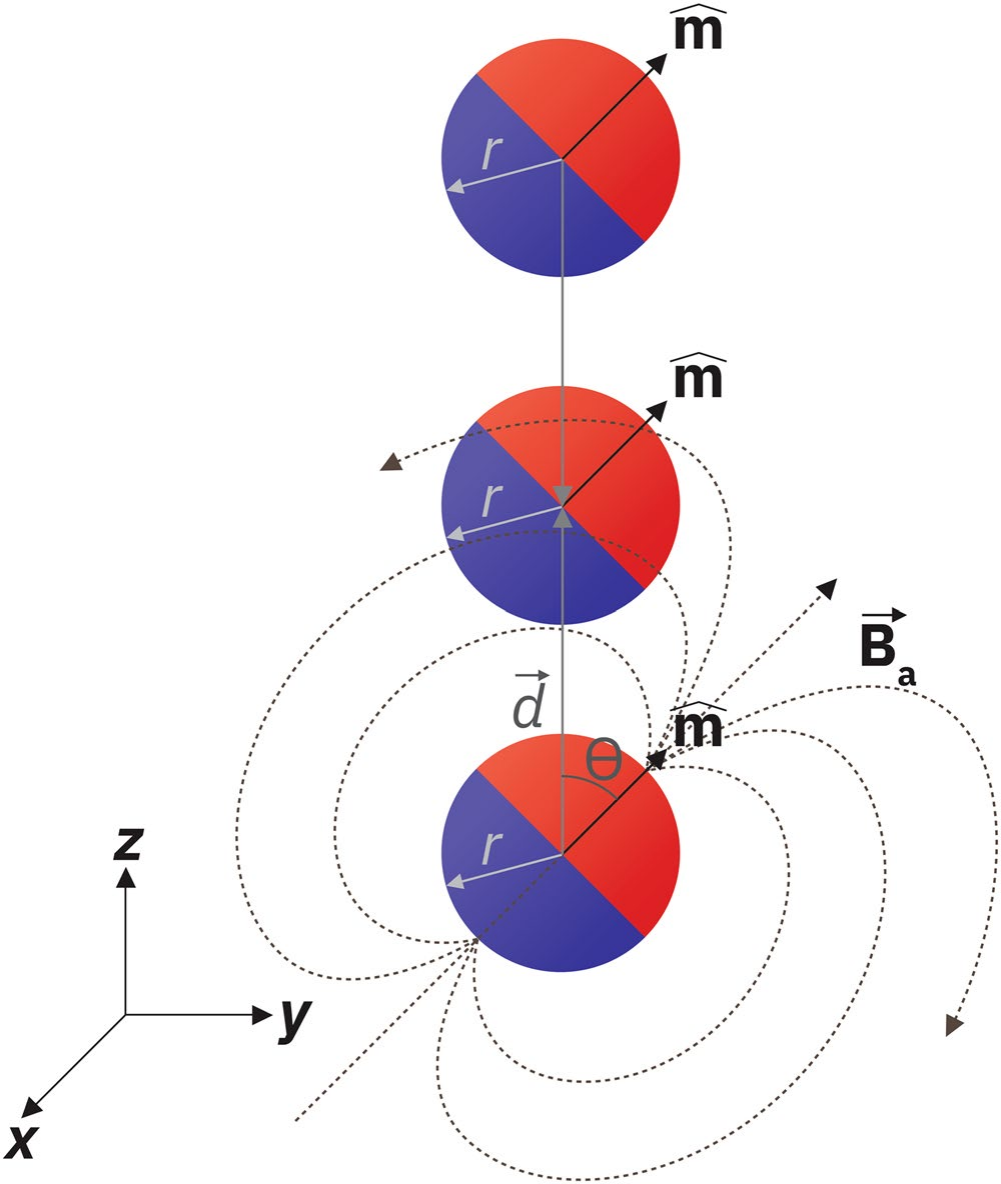
Magnetic field experienced by each magnet due to the adjacent magnet.

For the magnetic pendulum array as shown in Fig. 2, the magnetic field from adjacent magnets can play a significant role as it is interacting with the pendulum’s motion. Considering the magnetic field generated at each pendulum by an adjacent pendulum that is placed along the longitudinal axis (*θ* = 0) at distance d as shown in Fig. 2, The magnetic field experienced by each magnet due to its adjacent magnets can be written in the form of a magnetic dipole field as:

13 $${\overrightarrow{B}}_{a}=\frac{{\mu }_{o}m}{4\pi }\frac{(3cos\theta \hat{d}-\hat{m})}{{d}^{3}}$$

The magnetic torque received by the pendulum can be written as:

14 $${\overrightarrow{T}}_{a}=-\,{B}_{ao}m\frac{3sin2\theta }{4}\hat{x}$$

where ${B}_{ao}$ is the radial magnetic field of the magnetic disc at the distance of d at zero swing angle, i.e.,

15 $${B}_{ao}=\frac{{\mu }_{o}m}{2\pi {d}^{3}}$$

In addition to the magnetic torque, a net force is often generated between two pendulums. However, it can be easily proven in a vertically aligned pendulum array that the net force from the adjacent pendulums cancel each other but the torque doubles at each pendulum. Therefore, one may write the total torque received by each pendulum in the array in the following form:

16 $$\overrightarrow{{{\rm{T}}}_{{\rm{a}}}}=-\,{B}_{ao}{M}_{s}V\frac{3sin2\theta }{4}\hat{x}$$

We use the small angle approximation ($sin\theta \approx \theta$) to simplify the resulting differential equation. The pendulum equation with both external and self-biasing now becomes:

17 $$\frac{{d}^{2}\theta }{d{t}^{2}}=-\,\frac{{\overrightarrow{T}}_{e}+{\overrightarrow{T}}_{a}}{I}\approx -\frac{2{M}_{s}({B}_{e}+3{B}_{ao})}{\rho {r}^{2}}\theta $$

**Figure 3:**
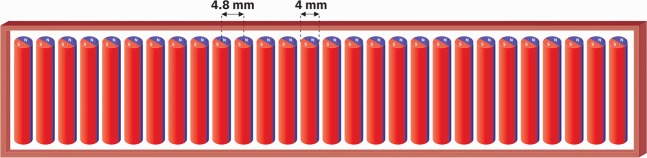
Schematic of a 28-element magnetic pendulum array consist of NdFeB magnets with 2 mm radius. Center to center distances between the magnets are 4.8 mm.

The equation can be solved to give *θ* as

18 $${\rm{\theta }}(t)={{\rm{\theta }}}_{{\rm{\max }}}\,\sin (\frac{1}{r}\sqrt{\frac{2{M}_{s}({B}_{e}+3{B}_{ao})}{\rho }}t)$$

which yields the pendulum oscillation frequency as:

19 $$f=\frac{1}{2\pi r}\sqrt{\frac{2{M}_{s}({B}_{e}+3{B}_{ao})}{\rho }}$$

where r is the radius of the cylinder, *M*_*s*_ is the magnetization density of the magnet in the pendulum, and *ρ* is the mass density of the magnet. It is to be noted that the frequency is inversely proportional to the radii of the magnets and directly proportional to the square root of the field strength and magnetization density. The desired frequency can be obtained by controlling these parameters. In our case, we have eliminated the need for a bulky external magnet by relying only on the self-bias field. The magnetic potential energy is transferred to the kinetic energy of the pendulums back and forth while transmitting a dynamic field outward. Assuming no external bias and NdFeB magnets of radius 2 mm and with center to center distance of 4.8 mm, we can calculate the expected oscillation frequency or angular frequency of such a pendulum array as shown in Fig. 3 from Eq. (19) as follows:

20 $$f=\frac{1}{2\pi r}\sqrt{\frac{6{M}_{s}{B}_{{a}_{o}}}{\rho }}$$

We have $r=2\,mm$, ${\mu }_{o}{M}_{s}=1.4\,T$, ${B}_{{a}_{o}}=2000\,G$ and $\rho =7500\,kg/{m}^{3}$. Substituting these values, we get

21 $$f=1062\,Hz$$

Thus, although we are in the ULF range with a frequency of 1062 Hz, to scale up in frequency and reach transmission frequencies in the kHz range, we need to reduce the radius of the magnets, the spacing between magnets or use stronger magnets to generate a higher self-bias field.

### Swing angle and quality factor calculation

The quality factor limited by friction loss can be estimated as follows: The mechanical energy associated with a magnet with a radius r, moment of inertia I, and rotating at an instantaneous angular frequency of $\Omega$, can be written as

22 $${W}_{ME}=\frac{1}{2}I{\Omega }^{2}=\frac{1}{2}{(\Omega r)}^{2}\rho V$$

The friction loss by bearings per cycle is given by

23 $${W}_{fr}=2\mu F{\theta }_{max}{r}_{b}$$

where *μ* is the co-efficient of friction, F is the residual force on the bearings due to the imbalance of the system, *r*_*b*_ is the radius of the bearing.

24 $$\Omega =\frac{{\rm{\partial }}\theta (t)}{{\rm{\partial }}t}={\theta }_{max}\cdot \omega cos(\omega t)$$

with $$\theta (t)={\theta }_{max}\,\sin (\omega t)$$

ω is the angular frequency of oscillation. The Q factor then turns out be

25 $$Q=2\pi \frac{{W}_{ME}}{{W}_{fr}}=\frac{3\pi {M}_{s}{B}_{{a}_{o}}{\theta }_{max}V{r}^{2}}{\mu F{r}_{b}}$$

Thus, by reducing the bearing radius and friction loss further, and increasing the volume fraction of the magnets, magnetic pendulum arrays should be able achieve extremely high Q factors.

### Near field radiation

The magnitude of the received magnetic field in the near field can be written as:

26 $$|{B}_{NF}|=\frac{2{\mu }_{o}{M}_{s}V}{4\pi {d}^{3}}sin{\theta }_{max}$$

where |*B*_*NF*_| is the magnitude of the magnetic near field, V is the effective magnetic volume, d is the distance, and *θ*_*max*_ is the maximum swing angle of the pendulum.

## Modelling and Simulation of the Magnetic Pendulum Array

**Figure 4:**
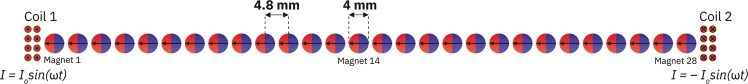
2D cross sectional model of Magnetic Pendulum Array in ANSYS Maxwell.

**Figure 5:**
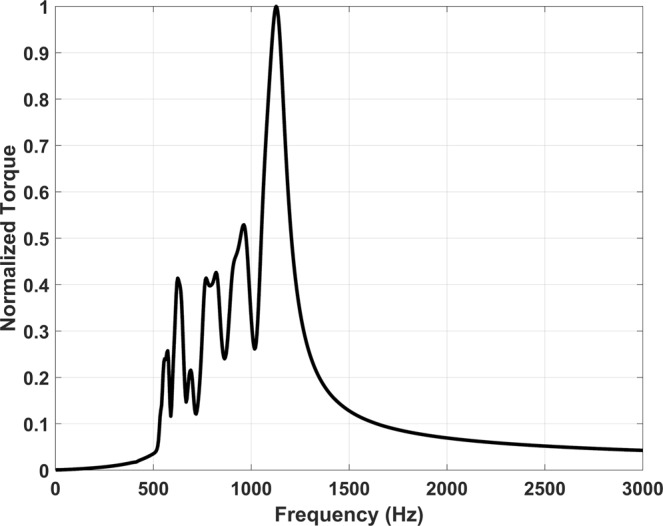
Torque on central magnet with wide band Gaussian excitation.

**Figure 6:**
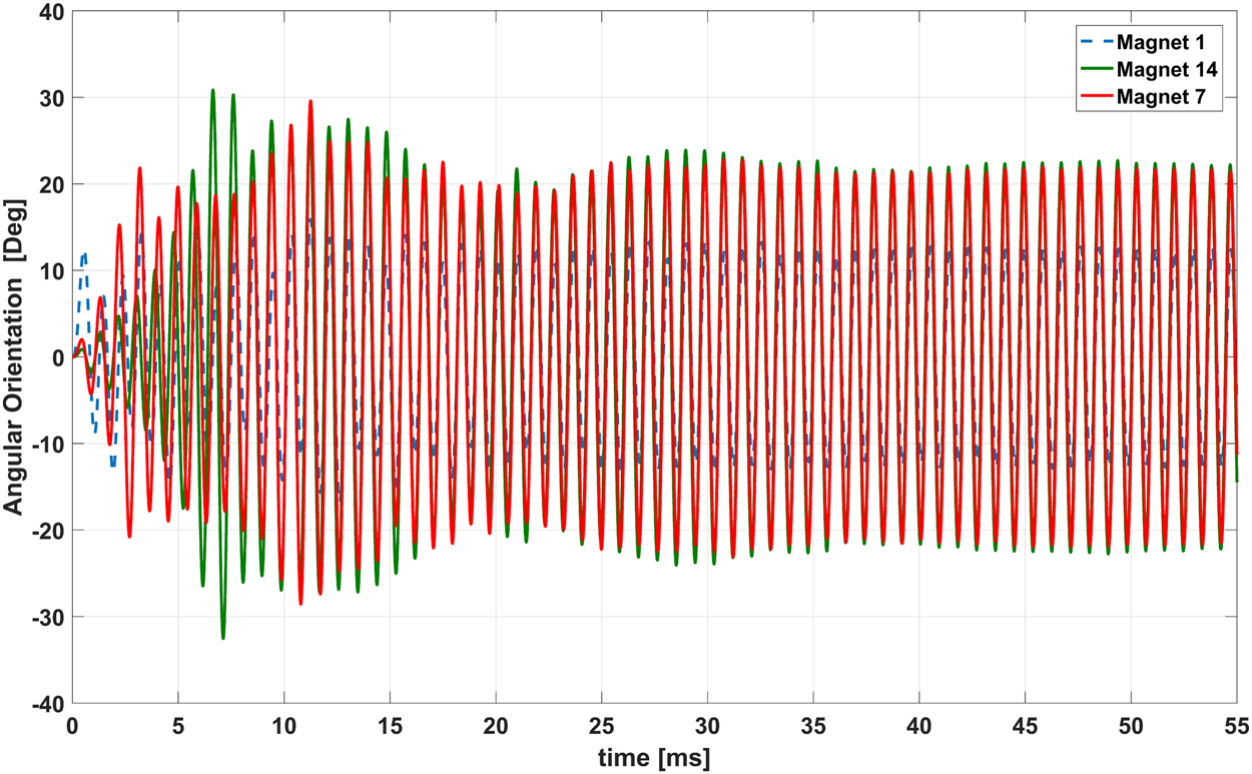
Simulated angular position of the magnets across time.

**Figure 7:**
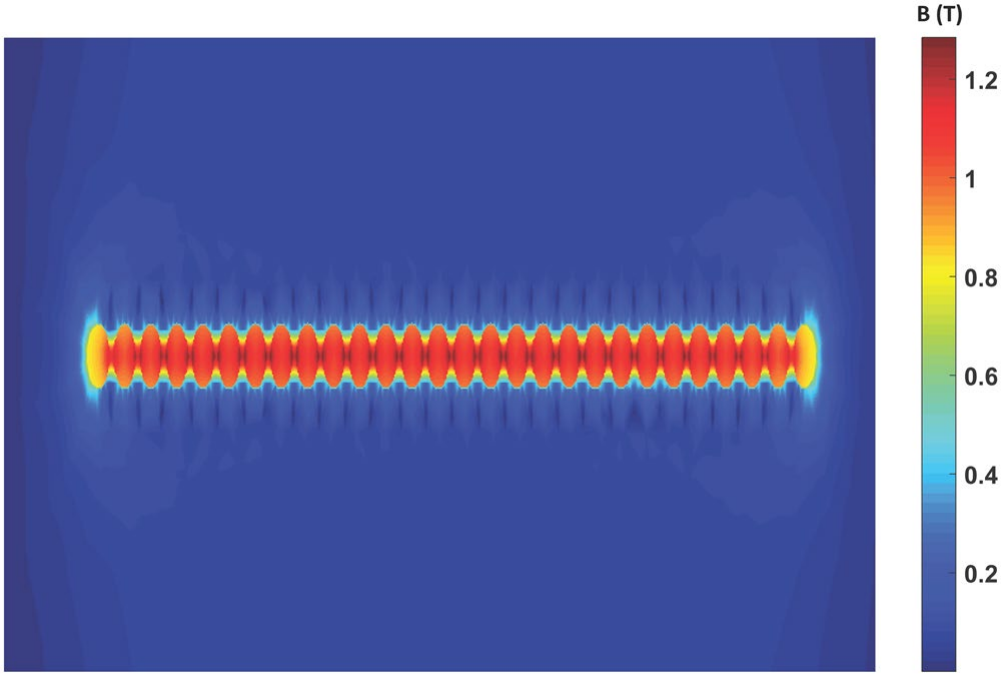
Magnetic field distribution. Observe the non-uniformity of the magnetic field across different magnets.

The magnetic pendulum array system consists of interactions between multiple physics such as Newton’s laws and Maxwell’s equations. It is important to accurately model such a system. For this purpose, we used ANSYS Maxwell which is a 3D full wave low frequency solver. ANSYS Maxwell is Finite Element Method (FEM)-based electromagnetic field simulation software for the design and analysis of electromagnetic and electromechanical devices. ANSYS Maxwell can simulate transient magnetic (time domain) fields caused by permanent magnets as function of time. Rotational motion effects, eddy current losses, and frictional and damping losses can be included in the simulation. 2D simulations are performed using ANSYS Maxwell to validate our concept and understand the interactions between multiple physical laws and their effect on system design. The model consists of an array of 28 cylindrical, diametrically magnetized magnets excited by a 55-turn coil as shown Fig. 4. The coil is first excited with a gaussian wideband excitation to determine the resonant modes of the magnetic pendulum array. The torque experienced by magnet closest to the center of the array is plotted across frequency in Fig. 5. The plot shows many distinct peaks corresponding to the different modes of the pendulum array. There is only one mode in which all the array elements oscillate in phase which is the mode at 1031 Hz. The magnetic pendulum array is then excited with this in phase mode frequency and the angular position of the edge magnets and the central magnet is plotted against time in Fig. 6. We can observe that it is an in-phase mode in which the magnets are oscillating in-phase with respect to each other, and so it is the most efficient for ULF transmission. It’s also clear from Fig. 6 that initially the magnets are not in phase and it takes time for the resonance to build up and the magnets to get aligned and oscillate in phase once the resonance is established. Also, in the in-phase mode not all the magnets have the same angular amplitude. This inconsistency can be attributed to the fact that the magnets do not see the same bias field. The edge magnets see lower bias than the inner ones and hence has the lower amplitude. This is further clarified from the plot of magnetic field distribution shown in Fig. 7.

## Prototype and Measurement Setup

**Figure 8:**
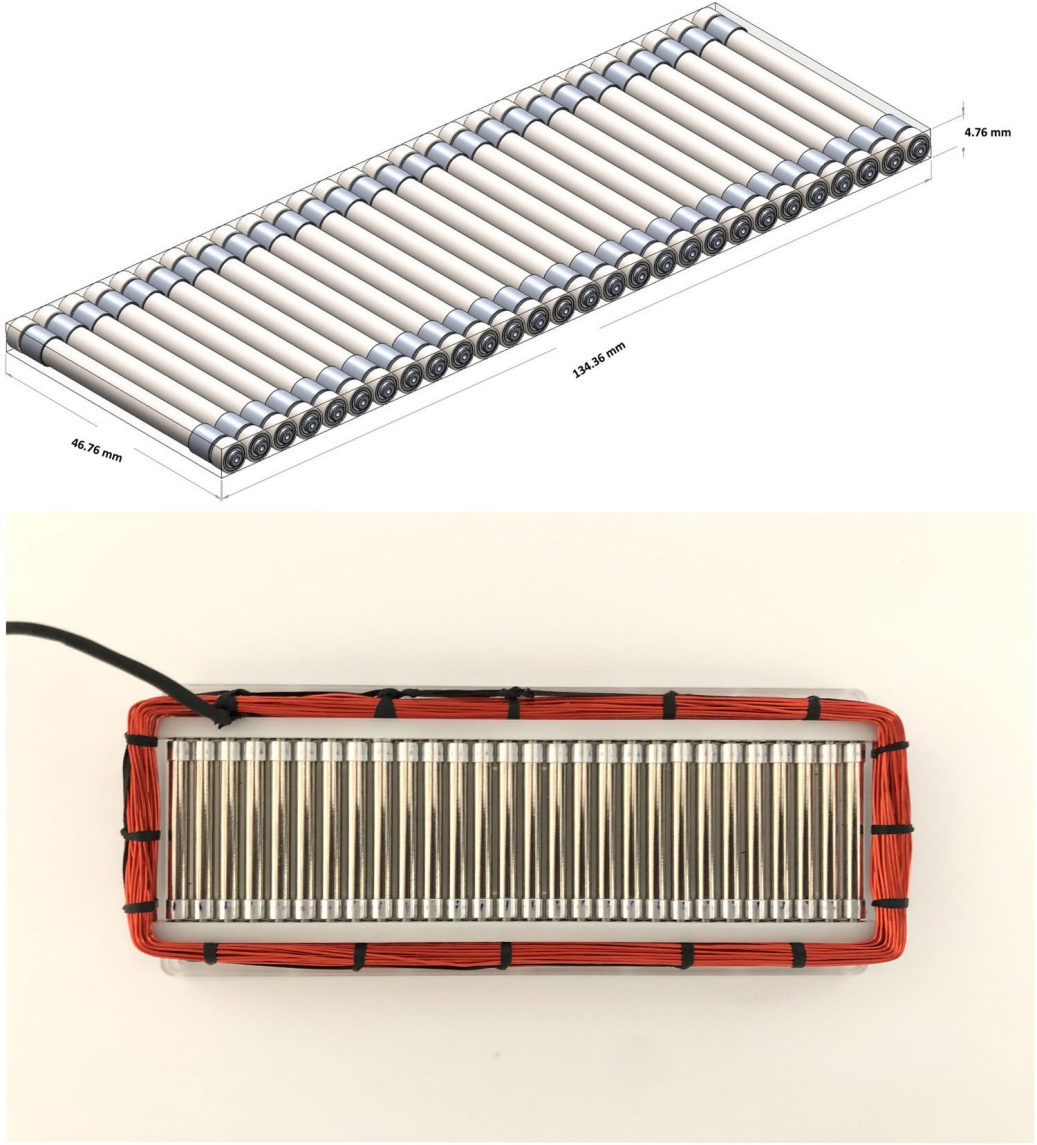
Magnetic pendulum array CAD model and prototype assembly.

**Figure 9:**
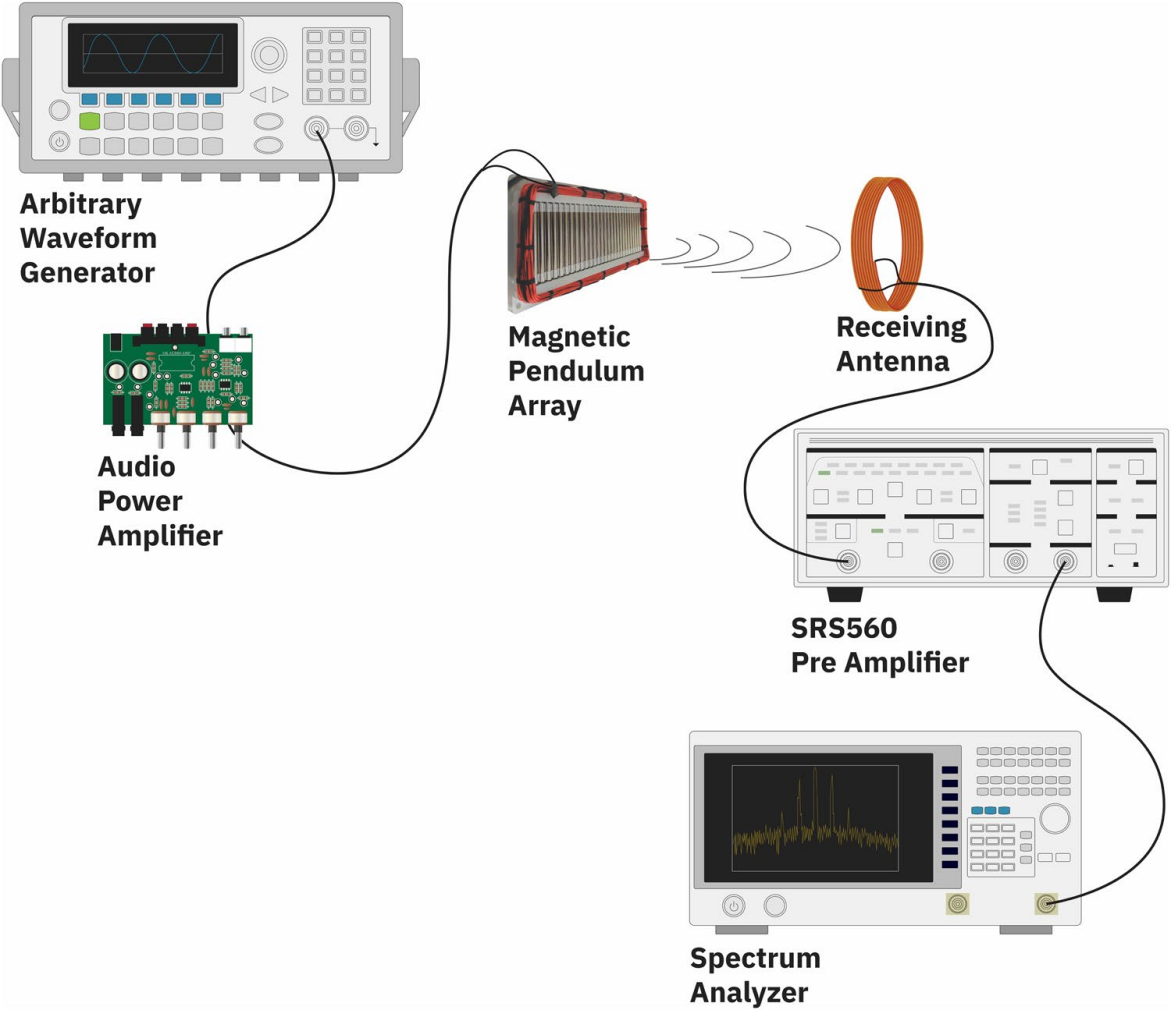
Schematic of the measurement setup.

A prototype of the magnetic pendulum array was fabricated and measured. The fabricated prototype is shown in Fig. 8 along with the CAD model. The magnetic pendulum array consists of 28 pendulum elements suspended by stainless steel ball bearings in a plastic housing. Each pendulum element is comprised of a 40 mm long diametrically magnetized cylindrical NdFeB N55 magnet having a 4 mm radius and is supported by aluminum bearing adapter sleeves on each end. The housing is made of plastic to prevent eddy current losses. A coil made of AWG26 copper wire with 55 turns is wrapped around the magnets to provide the excitation field. The sinusoidal input signal is generated from a signal generator and amplified using an audio power amplifier before being fed to the Magnetic Pendulum Array. The receiver is a 45-turn loop antenna of radius 26 cm, and a spectrum analyzer is used to observe the received signal. The measurement setup is diagrammatically represented in Fig. 9.

### Near field transmission measurement

**Figure 10:**
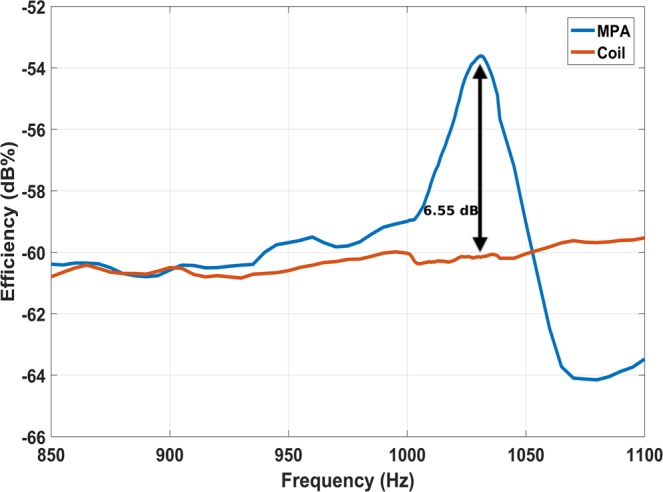
Transmission efficiency of magnetic pendulum array compared to that of a bare coil.

**Figure 11:**
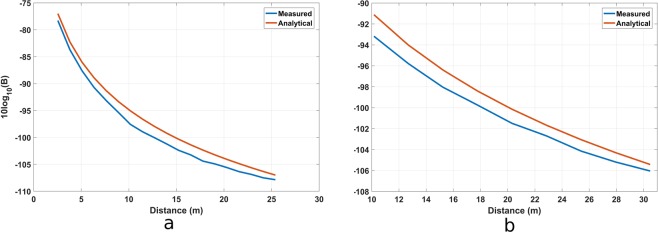
Received power vs distance using a 45-turn loop antenna as receiver. Measurements were done at 1031 Hz. Input power to the coils are (**a**) 0.6 W (**b**) 1.9 W.

**Figure 12:**
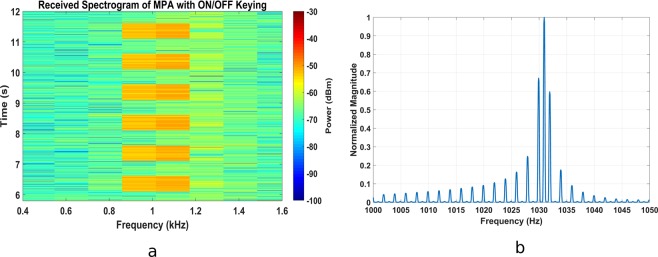
Data transmission test with OOK (**a**). Received signal spectrogram (**b**). Received signal spectrum.

The transmission efficiency of the magnetic pendulum array as compared to a bare coil is plotted in Fig. 10. In general, the efficiency of coupling increases with the frequency for both cases as a greater quality factor is obtained for the coil at higher frequency which helps to increase the power coupled through nearfield^17^. The efficiency is maximum in the in-phase mode at 1031 Hz, as expected. The transmission efficiency of magnetic pendulum array is about 7 dB higher than a bare coil at 1031 Hz. The spiking of the efficiency at resonance can be explained by the fact that, more power is coupled to the pendulum array versus that is coupled to the coil. A range test was performed at the resonance frequency, results of which are shown in Fig. 11. The transmitted power into the coil loaded with the magnetic pendulum array are 0.6 W and 1.9 W for Fig. 11a,b, respectively. The measured results are compared with the analytical results derived from the near field equation shown in (26) and a good agreement is observed. A range of 25 m and 30 m were obtained on the receiver end for 0.6 W and 1.9 W input powers as shown in Fig. 11. A data transmission test was performed at 2 bit per second (bps) modulation rate with On-Off Keying (OOK) modulation scheme. The test was performed indoors with the receiver at 3 m from transmitter. The received spectrogram is shown in Fig. 12(a) and the received spectrum is shown in Fig. 12(b). Even though the data rate is small, it demonstrates that data can be successfully transmitted using magnetic pendulum arrays. At higher Q factors Direct Antenna Modulation (DAM)^13^ can be implemented with more complex modulation schemes such as Binary Frequency Shift Keying (BFSK) to achieve higher data rates.

### Equivalent circuit model

**Figure 13:**
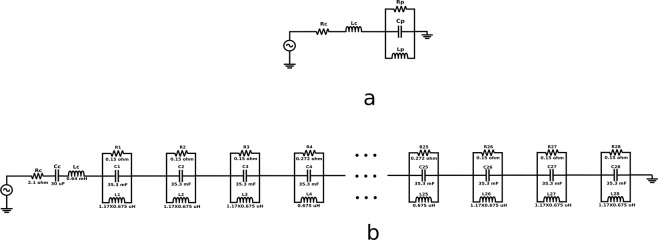
Equivalent circuit model for Magnetic Pendulum Array (**a**). Equivalent circuit model for one element (**b**). Equivalent circuit model for 28-element Magnetic Pendulum Array. Observe that the inductance on the edge elements is higher than in the middle accounting for non-uniform bias and resistance is much lower on the edges, indicating a higher loss and lower Q.

In this section, we develop an equivalent circuit model based on the physics of coupling between the coil and magnets. Circuit models can help us understand the system in an intuitive way and are great tools to quickly verify some of the properties of the system. The magnetic pendulum array can be modeled as a parallel RLC circuit, as shown in Fig. 13(a). The resistance of the coil is modeled as ${R}_{c}$ and the inductance of the coil is modeled as ${L}_{c}$. The resistance ${R}_{p}$ represents mainly the friction in the bearings. The capacitor ${C}_{p}$ represents the kinetic energy of the pendulum, and the inductance ${L}_{p}$ represents the magnetic potential energy stored in the flux associated with the pendulum. The magnetic field from the coil that turns the magnets can be modeled as the current in the circuit, and the EMF generated in the coil by the oscillating magnets can be modeled as voltage. Both, a series and a parallel resonant mode can be observed in the pendulum array. The parallel resonant mode ${\omega }_{p}$ can be written as

27 $${{\rm{\omega }}}_{{\rm{p}}}=\frac{1}{\sqrt{{L}_{p}{C}_{p}}}$$

At resonance, the imaginary part of the sum of the impedances can be equated to zero as

28 $$Imag\,({j{\rm{\omega }}}_{{\rm{s}}}{L}_{c}+\frac{1}{\frac{1}{j{\omega }_{s}{L}_{p}}+j{\omega }_{s}{C}_{p}+\frac{1}{{R}_{p}}})=0$$

Assuming the system Q factor to be high, we can neglect the frictional and mechanical losses and ignore the $\frac{1}{{R}_{p}}$ term. Further simplification for the series resonance ${\omega }_{s}$ leads to

29 $${{\rm{\omega }}}_{{\rm{s}}}=\sqrt{\frac{{L}_{C}+{L}_{P}}{{L}_{C}{L}_{P}{C}_{P}}}$$

The model can be extended to an array by cascading the single element to include the effect of non-uniform bias on the magnets at both ends, as shown in Fig. 13(b). Capacitor *C*_*c*_ is used to compensate for the inductance of the coil and match the impedance. For the 28-element magnetic pendulum array as shown in Fig. 3. the circuit parameters are calculated as follows,

$$\begin{array}{c}{R}_{c}=2.1\,\Omega ,\,{C}_{c}=30\,{\rm{\mu }}F,\,{L}_{c}=0.64\,{\rm{mH}},\\ {R}_{1}={R}_{2}={R}_{3}={R}_{26}={R}_{27}={R}_{28}=0.15\,\Omega ,\,{R}_{4}={R}_{5}=\cdots ={R}_{25}=0.272\,\Omega \\ {C}_{1}={C}_{2}=\cdots ={C}_{28}=35.3\,{\rm{mF}}\\ {L}_{1}={L}_{2}={L}_{3}={L}_{26}={L}_{27}={L}_{28}=0.79\,\mu H,\,{L}_{4}={L}_{5}=\cdots ={L}_{25}=0.675\,\mu H\end{array}$$

When the impedance of the circuit is plotted across frequency which is presented in the following section, sharp resonance is observed at around 1031 Hz consistent with our predicted value of resonance frequency. The circuit model clearly demonstrates the advantage of using mechanical antennas and the system resonance for ULF transmission. At resonance, the current through the coil (represented by ${R}_{c}$ and ${L}_{c}$) is significantly reduced, thereby reducing the ohmic losses present in the system. The circuit model is not a complete description of the system as it does not consider the time varying nature of the flux and the non-linearities inherently present in this system. But the circuit model is still of great help in visualizing the system, verifying the predicted values of the resonance frequency, and estimating the Q factor of the system. The Q can be estimated as follows:

30 $$Q=R\sqrt{\frac{C}{L}}=0.272\sqrt{\frac{35.3\times {10}^{-3}}{0.675\times {10}^{-6}}}=62.2$$

**Figure 14:**
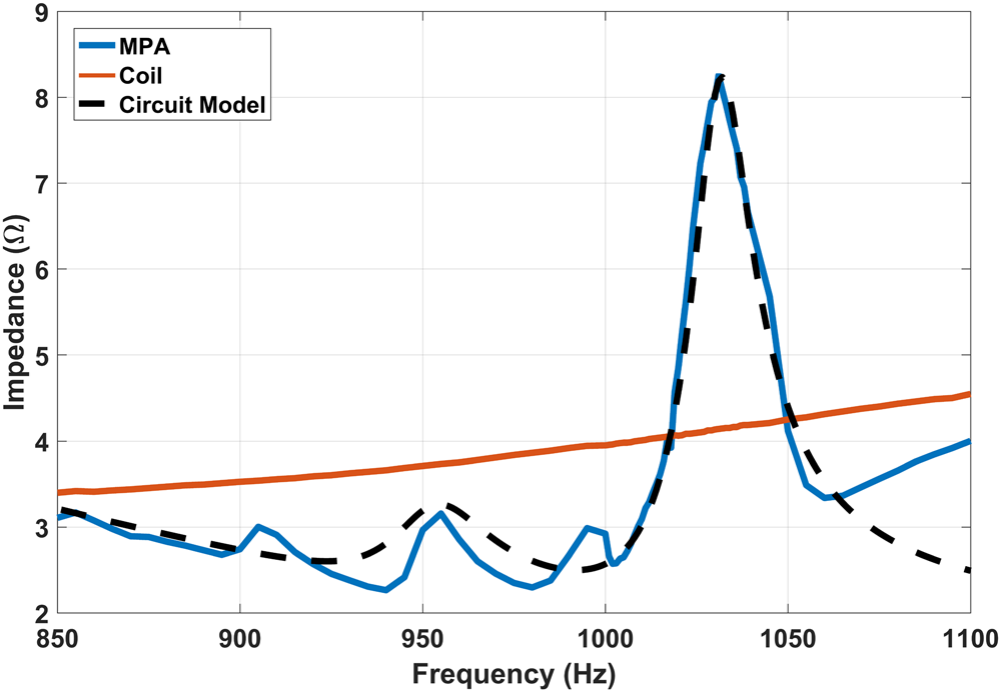
Measured impedance and predicted impedance from circuit model versus frequency.

Figure 13(b) shows the improved circuit model and Fig. 14 shows input impedance of the pendulum array prototype, coil and the circuit model plotted across frequency. The peaking of the input impedance indicates a greater portion of the input power is coupled to the pendulum system while the power consumption caused by the Ohmic resonance of coil is relatively reduced. Note that not all the modes were predicted by the circuit model due to the fact the mutual coupling among the pendulum elements are not included in the model demonstrating the superiority and completeness of the full wave model. The estimated Q factor at the in-phase mode is around 62.2 as previously demonstrated using the circuit model. To our knowledge, this is the first time such a high Q factor has been achieved in a mechanical antenna system using magnetic dipoles at ULF, demonstrating the potential of magnetic pendulum arrays for efficient ULF transmission.

## Discussions

An innovative system with a high Q factor for efficient ULF generation and transmission, which utilizes mechanical resonance was presented. A proof of concept demonstration was shown at 1031 Hz along with 2 bps modulation. It was observed that the efficiency of the pendulum array is about 7 dB higher than the bare coils. The current system can be scaled up in frequency by tighter packing of magnets, higher Q factors can be achieved by improving the mechanical system and resulting more efficient radiation. The results presented here demonstrate the feasibility of using high-Q magnetic pendulum arrays for efficiently transmitting Ultra Low Frequencies. We believe a host of other applications beyond underwater communications, such as wireless power transfer and underground localization can benefit from Magnetic Pendulum Arrays.
